# Supplementary material for: Relationships between Maternal Dietary Patterns and Blood Lipid Levels during Pregnancy: A Prospective Cohort Study in Shanghai, China
Source: Int J Environ Res Public Health. 2021 Apr 1;18(7):3701. doi: 10.3390/ijerph18073701 (PMC8036959; doi:10.3390/ijerph18073701)
Supplement: Supplementary file 1 [file ijerph-18-03701-s001.pdf]

Supplementary materials

Table S1. Blood lipid levels during pregnancy according to adherence to dietary patterns identified by PCA.

Outcomes	Quartiles of dietary pattern scores				*P* value
	Q1	Q2	Q3	Q4	
**Tubers-fruit-vegetables**					
**TG (mmol/L)**					
First trimester	1.23 (0.96, 1.49)	1.17 (0.94, 1.57)	1.24 (0.96, 1.57)	1.19 (0.94, 1.46)	0.778
Second trimester	2.14 (1.71, 2.90)	2.31 (1.82, 2.84)	2.19 (1.78, 2.79)	2.17 (1.75, 2.72)	0.614
Third trimester	2.94 (2.41, 3.77)	3.08 (2.50, 3.81)	2.95 (2.38, 3.67)	2.98 (2.32, 3.66)	0.336
**TC (mmol/L)**					
First trimester	4.61 (4.20, 5.07)	4.47 (4.02, 5.09)	4.45 (4.00, 5.01)	4.38 (3.95, 4.72)	**0.002**
Second trimester	6.29 (5.71, 7.11)	6.06 (5.51, 6.78)	6.11 (5.53, 6.76)	5.98 (5.33, 6.53)	**<0.001**
Third trimester	6.67 (6.04, 7.55)	6.44 (5.89, 7.19)	6.43 (5.68, 7.27)	6.25 (5.48, 7.03)	**<0.001**
**HDL-C (mmol/L)**					
First trimester	1.10 (1.00, 1.21)	1.07 (0.96, 1.22)	1.06 (0.95, 1.21)	1.04 (0.94, 1.13)	**0.001**
Second trimester	1.53 (1.40, 1.75)	1.48 (1.35, 1.66)	1.49 (1.36, 1.67)	1.46 (1.30, 1.62)	**0.001**
Third trimester	1.62 (1.45,1.83)	1.56 (1.42, 1.74)	1.56 (1.38, 1.76)	1.52 (1.29, 1.71)	**<0.001**
**LDL-C (mmol/L)**					
First trimester	2.97 (2.72, 3.23)	2.91 (2.70, 3.25)	2.90 (2.67, 3.20)	2.85 (2.61, 3.06)	**0.003**
Second trimester	4.06 (3.75, 4.46)	3.94 (3.56, 4.33)	3.90 (3.58, 4.33)	3.86 (3.50, 4.19)	**<0.001**
Third trimester	4.16 (3.78, 4.68)	4.07 (3.70, 4.46)	4.04 (3.60, 4.49)	3.97 (3.47, 4.40)	**<0.001**
**Beans-fungi-algae**					
**TG (mmol/L)**					
First trimester	1.23 (0.96, 1.57)	1.21 (0.96, 1.49)	1.18 (0.94, 1.51)	1.20 (0.94, 1.53)	0.826
Second trimester	2.21 (1.80, 2.87)	2.22 (1.77, 2.73)	2.14 (1.70, 2.78)	2.25 (1.81, 2.89)	0.570
Third trimester	3.07 (2.44, 3.89)	2.99 (2.41, 3.61)	2.84 (2.36, 3.62)	3.03 (2.45, 3.77)	0.259
**TC (mmol/L)**					
First trimester	4.52 (4.10, 5.10)	4.45 (4.01, 4.89)	4.41 (4.01, 4.91)	4.49 (3.96, 4.95)	0.261
Second trimester	6.23 (5.50, 6.96)	6.09 (5.61, 6.67)	6.06 (5.50, 6.84)	6.04 (5.54, 6.80)	0.315
Third trimester	6.58 (5.90, 7.36)	6.41 (5.83, 7.11)	6.47 (5.61, 7.27)	6.47 (5.72, 7.37)	0.444
**HDL-C (mmol/L)**					
First trimester	1.08 (0.98, 1.22)	1.06 (0.96, 1.17)	1.06 (0.96, 1.18)	1.07 (0.94, 1.18)	0.213
Second trimester	1.51 (1.36, 1.72)	1.49 (1.36, 1.64)	1.50 (1.34, 1.66)	1.49 (1.34, 1.67)	0.279
Third trimester	1.57 (1.39, 1.81)	1.54 (1.40, 1.70)	1.56 (1.33, 1.74)	1.58 (1.37, 1.79)	0.427
**LDL-C (mmol/L)**					
First trimester	2.93 (2.71, 3.24)	2.91 (2.65, 3.16)	2.90 (2.66, 3.13)	2.92 (2.61, 3.17)	0.424
Second trimester	4.02 (3.59, 4.43)	3.90 (3.65, 4.20)	3.90 (3.54, 4.32)	3.90 (3.60, 4.36)	0.290
Third trimester	4.05 (3.68, 4.53)	4.03 (3.68, 4.44)	4.07 (3.51, 4.45)	4.04 (3.58, 4.48)	0.600

**Fish-shrimps**					
**TG (mmol/L)**					
First trimester	1.24 (0.98, 1.58)	1.20 (0.99, 1.53)	1.17 (0.93, 1.52)	1.22 (0.94, 1.48)	0.507
Second trimester	2.21 (1.84, 2.87)	2.19 (1.78, 2.78)	2.21 (1.75, 2.79)	2.19 (1.70, 2.79)	0.913
Third trimester	3.01(2.51, 3.70)	2.99 (2.37, 3.74)	2.93 (2.43, 3.73)	3.01 (2.41, 3.71)	0.960
**TC (mmol/L)**					
First trimester	4.38 (3.96, 4.88)	4.47 (4.05, 4.92)	4.50 (4.02, 5.11)	4.56 (4.15, 5.01)	0.147
Second trimester	6.09 (5.51, 6.75)	6.02 (5.52, 6.62)	6.08 (5.46, 6.97)	6.36 (5.62, 6.83)	0.138
Third trimester	6.29 (5.70, 7.08)	6.40 (5.68, 7.17)	6.55 (5.82, 7.29)	6.56 (5.93, 7.36)	0.215
**HDL-C (mmol/L)**					
First trimester	1.04 (0.94, 1.16)	1.06 (0.96, 1.18)	1.08 (0.96, 1.20)	1.09 (0.99, 1.22)	0.111
Second trimester	1.48 (1.34, 1.65)	1.45 (1.35, 1.61)	1.51 (1.33, 1.70)	1.55 (1.38, 1.69)	0.057
Third trimester	1.52 (1.36, 1.72)	1.56 (1.37, 1.72)	1.59 (1.40, 1.80)	1.58 (1.42, 1.80)	0.100
**LDL-C (mmol/L)**					
First trimester	2.85 (2.61, 3.12)	2.91 (2.67, 3.17)	2.95 (2.67, 3.26)	2.94 (2.73, 3.20)	0.086
Second trimester	3.89 (3.57, 4.31)	3.81 (3.60, 4.26)	3.94 (3.55, 4.43)	4.02 (3.65, 4.35)	0.083
Third trimester	3.99 (3.55, 4.41)	4.03 (3.58, 4.44)	4.13 (3.64, 4.52)	4.09 (3.74, 4.57)	0.200
**Refined grains-red meat-organs**					
**TG (mmol/L)**					
First trimester	1.18 (0.94, 1.49)	1.20 (0.94, 1.46)	1.18 (0.94, 1.58)	1.24 (1.00, 1.54)	0.332
Second trimester	2.20 (1.74, 2.81)	2.17 (1.71, 2.78)	2.21 (1.84, 2.89)	2.22 (1.78, 2.80)	0.455
Third trimester	2.97 (2.40, 3.70)	3.04 (2.45, 3.71)	2.98 (2.43, 3.84)	2.96 (2.42, 3.67)	0.886
**TC (mmol/L)**					
First trimester	4.49 (4.10, 4.96)	4.39 (3.93, 4.90)	4.49 (4.02, 5.00)	4.51 (4.11, 5.01)	0.105
Second trimester	6.15 (5.54, 6.81)	6.06 (5.48, 6.80)	6.09 (5.48, 6.75)	6.09 (5.61, 6.85)	0.787
Third trimester	6.47 (5.80, 7.30)	6.46 (5.73, 7.19)	6.38 (5.78, 7.22)	6.55 (5.79, 7.25)	0.711
**HDL-C (mmol/L)**					
First trimester	1.07 (0.98, 1.19)	1.03 (0.94, 1.18)	1.08 (0.95, 1.20)	1.09 (0.97, 1.20)	0.096
Second trimester	1.52 (1.36, 1.66)	1.49 (1.34, 1.67)	1.49 (1.34, 1.68)	1.49 (1.37, 1.67)	0.775
Third trimester	1.58 (1.39, 1.77)	1.56 (1.39, 1.76)	1.53 (1.36, 1.73)	1.58 (1.40, 1.75)	0.566
**LDL-C (mmol/L)**					
First trimester	2.92 (2.68, 3.19)	2.83 (2.61, 3.15)	2.92 (2.66, 3.17)	2.96 (2.71, 3.24)	0.050
Second trimester	3.95 (3.63, 4.35)	3.93 (3.50, 4.30)	3.91 (3.60, 4.32)	3.90 (3.65, 4.41)	0.771
Third trimester	4.11 (3.66, 4.50)	4.05 (3.64, 4.49)	3.95 (3.58, 4.47)	4.09 (3.63, 4.49)	0.505
**Confectionery-sugared beverages**					
**TG (mmol/L)**					
First trimester	1.20 (0.94, 1.52)	1.22 (0.94, 1.55)	1.24 (0.97, 1.54)	1.19 (0.94, 1.51)	0.827
Second trimester	2.12 (1.77, 2.60)	2.26 (1.73, 2.87)	2.18 (1.81, 2.89)	2.24 (1.74, 2.89)	0.490
Third trimester	2.89 (2.32, 3.72)	3.04 (2.46, 3.97)	2.96 (2.40, 3.61)	3.00 (2.44, 3.72)	0.464
**TC (mmol/L)**					

First trimester	4.43 (3.96, 4.97)	4.46 (4.11, 5.01)	4.49 (4.07, 4.98)	4.51 (4.02, 4.96)	0.756
Second trimester	6.03 (5.39, 6.64)	6.15 (5.52, 6.76)	6.06 (5.55, 6.84)	6.19 (5.60, 6.90)	0.272
Third trimester	6.38 (5.53, 7.08)	6.46 (5.83, 7.36)	6.54 (5.93, 7.27)	6.49 (5.71, 7.24)	0.171
**HDL-C (mmol/L)**					
First trimester	1.05 (0.95, 1.18)	1.07 (0.98, 1.18)	1.07 (0.97, 1.21)	1.07 (0.96, 1.19)	0.675
Second trimester	1.48 (1.31, 1.62)	1.50 (1.36, 1.67)	1.50 (1.37, 1.67)	1.52 (1.36, 1.70)	0.234
Third trimester	1.54 (1.32, 1.73)	1.58 (1.40, 1.78)	1.57 (1.42, 1.77)	1.58 (1.38, 1.78)	0.162
**LDL-C (mmol/L)**					
First trimester	2.87 (2.64, 3.16)	2.91 (2.69, 3.20)	2.91 (2.67, 3.24)	2.94 (2.65, 3.16)	0.765
Second trimester	3.88 (3.54, 4.20)	3.94 (3.60, 4.35)	3.93 (3.60, 4.34)	4.01 (3.65, 4.41)	0.290
Third trimester	4.02 (3.51, 4.40)	4.04 (3.67, 4.53)	4.09 (3.66, 4.50)	4.06 (3.62, 4.51)	0.105

TC total cholesterol, HDL-C high-density lipoprotein cholesterol, LDL-C low-density lipoprotein cholesterol, TG triacylglycerol.

Table S2. The contribution of nutrients and food intake to the association between dietary pattern scores and blood lipid levels during pregnancy

**Outcomes**	**Tubers-fruit-vegetables** β (95 %CI)	***P* value**	**Beans-fungi-algae** β (95 %CI)	***P* value**	**Fish-shrimps** β (95 %CI)	***P* value**	**Refined grains-red meat-organs** β (95 %CI)	***P* value**	**Confectionery-sugared beverages** β (95 %CI)	***P* value**
**First trimester**										
**Adjusted model**										
TC	-0.09 (-0.14, -0.04)	**0.001**	-0.02(-0.07, 0.03)	0.481	0.09 (0.04, 0.14)	**0.001**	-0.03(-0.08, 0.02)	0.238	0.02(-0.03, 0.08)	0.351
HDL-C	-0.02(-0.04, -0.01)	**<0.001**	-0.01 (-0.02, 0.01)	0.436	0.02 (0.01, 0.04)	**<0.001**	-0.01(-0.02, 0.01)	0.276	0.01 (-0.01,0.02)	0.209
LDL-C	-0.05 (-0.08,-0.02)	**0.001**	-0.02(-0.05, 0.01)	0.170	0.04 (0.01, 0.07)	**0.012**	-0.01(-0.04, 0.02)	0.617	0.01(-0.02, 0.04)	0.501
TG	-0.02 (-0.06, 0.03)	0.551	0.03 (-0.02, 0.08)	0.200	-0.02 (-0.07, 0.03)	0.354	-0.02 (-0.06,0.03)	0.539	-0.02 (-0.07, 0.03)	0.393
**Adjusted model +CHO (En%)**										
TC	-0.07 (-0.13, -0.02)	**0.010**	-0.03 (-0.08, 0.02)	0.258	0.05 (0.00, 0.11)	0.059	-0.04 (-0.09, 0.02)	0.160	0.03 (-0.02, 0.09)	0.217
HDL-C	-0.02 (-0.03, -0.01)	**0.005**	-0.01 (-0.02, 0.01)	0.219	0.02 (0.00, 0.03)	**0.032**	-0.01 (-0.02, 0.00)	0.184	0.01 (0.00, 0.02)	0.115
LDL-C	-0.04 (-0.07, -0.01)	**0.018**	-0.02 (-0.05, 0.01)	0.150	0.03 (0.00, 0.07)	**0.032**	-0.02 (-0.05, 0.01)	0.158	0.02 (-0.01, 0.05)	0.215
TG	-0.02 (-0.07, 0.03)	0.361	0.03 (-0.01, 0.08)	0.139	-0.01 (-0.06, 0.05)	0.804	-0.01 (-0.06, 0.04)	0.616	-0.02 (-0.07, 0.02)	0.318
**Adjusted model +Pro (En%)**										
TC	-0.08 (-0.13, -0.03)	**0.003**	-0.03 (-0.08, 0.02)	0.194	0.04 (-0.03, 0.10)	0.283	-0.03 (-0.08, 0.02)	0.291	0.04 (-0.01, 0.09)	0.134
HDL-C	-0.02 (-0.03, -0.01)	**0.001**	-0.01 (-0.02, 0.00)	0.166	0.01 (-0.01, 0.03)	0.190	-0.01 (-0.02, 0.01)	0.336	0.01 (0.00, 0.03)	0.068
LDL-C	-0.04 (-0.07, -0.01)	**0.007**	-0.02 (-0.05, 0.00)	0.097	0.02 (-0.02, 0.06)	0.250	-0.02 (-0.05, 0.01)	0.282	0.02 (-0.01, 0.05)	0.121
TG	-0.02 (-0.07, 0.03)	0.469	0.04 (-0.01, 0.08)	0.138	0.00 (-0.06, 0.06)	0.916	-0.02 (-0.06, 0.03)	0.507	-0.03 (-0.08, 0.02)	0.289

**Adjusted model +Pro /CHO(En%)**										
TC	-0.08 (-0.13, -0.02)	**0.006**	-0.03 (-0.08, 0.02)	0.187	0.03 (-0.03, 0.10)	0.300	-0.03 (-0.08, 0.02)	0.300	0.04 (-0.01, 0.09)	0.142
HDL-C	-0.02 (-0.03, -0.01)	**0.003**	-0.01 (-0.02, 0.00)	0.161	0.01 (-0.01, 0.03)	0.161	-0.01 (-0.02, 0.01)	0.258	0.01 (0.00, 0.03)	0.073
LDL-C	-0.04 (-0.07, -0.01)	**0.013**	-0.02 (-0.05, 0.01)	0.100	0.02 (-0.01, 0.06)	0.223	-0.02 (-0.05, 0.01)	0.213	0.02 (-0.01, 0.05)	0.137
TG	-0.02 (-0.07, 0.03)	0.392	-0.02 (-0.07, 0.03)	0.119	0.00 (-0.06, 0.06)	0.931	-0.01 (-0.06, 0.03)	0.550	-0.01 (-0.06, 0.03)	0.550
**Adjusted model + total fat**										
TC	-0.08 (-0.13, -0.03)	**0.003**	-0.02 (-0.07, 0.03)	0.376	0.08 (0.03, 0.13)	**0.002**	-0.03 (-0.09, 0.02)	0.197	0.03 (-0.02, 0.08)	0.306
HDL-C	-0.02 (-0.03, -0.01)	**0.001**	-0.01 (-0.02, 0.01)	0.330	0.02 (0.01, 0.03)	**0.001**	-0.01 (-0.02, 0.00)	0.227	0.01 (0.00, 0.02)	0.175
LDL-C	-0.04 (-0.07, -0.01)	**0.006**	-0.02 (-0.04, 0.01)	0.230	0.05 (0.02, 0.08)	**0.001**	-0.02 (-0.05, 0.01)	0.195	0.02 (-0.01, 0.04)	0.298
TG	-0.02 (-0.07, 0.03)	0.444	0.03 (-0.01, 0.08)	0.167	-0.02 (-0.07, 0.03)	0.439	-0.01 (-0.06, 0.03)	0.583	-0.02 (-0.07, 0.03)	0.365
**Adjusted model + dietary cholesterol**										
TC	-0.09 (-0.14, -0.04)	**0.001**	-0.02 (-0.07, 0.03)	0.454	0.04 (-0.03, 0.11)	0.255	-0.03 (-0.09, 0.02)	0.195	0.02 (-0.03, 0.07)	0.493
HDL-C	-0.02 (-0.04, -0.01)	**<0.001**	-0.01 (-0.02, 0.01)	0.409	0.01 (-0.01, 0.03)	0.183	-0.01 (-0.02, 0.01)	0.225	0.01 (-0.01, 0.02)	0.319
LDL-C	-0.05 (-0.08, -0.02)	**0.002**	-0.02 (-0.04, 0.01)	0.273	0.03 (-0.01, 0.07)	0.201	-0.02 (-0.05, 0.01)	0.186	0.01 (-0.02, 0.04)	0.472
TG	-0.01 (-0.06, 0.03)	0.553	0.03 (-0.02, 0.08)	0.192	0.00 (-0.06, 0.07)	0.981	-0.01 (-0.06, 0.03)	0.581	-0.02 (-0.07, 0.03)	0.471
**Second trimester**										

En%, percentage energy;

**Adjusted model**										
TC	-0.15 (-0.22, -0.07)	**<0.001**	-0.04 (-0.11, 0.03)	0.311	0.11 (0.04, 0.18)	**0.003**	-0.03(-0.10, 0.05)	0.501	0.08 (0.01, 0.16)	**0.028**
HDL-C	-0.03 (-0.05, -0.01)	**0.001**	-0.01(-0.03, 0.01)	0.164	0.03 (0.01, 0.05)	**0.001**	-0.01 (-0.03, 0.01)	0.575	0.02 (0.01, 0.04)	**0.015**
LDL-C	-0.08 (-0.12, -0.03)	**0.001**	-0.02 (-0.07, 0.02)	0.259	0.07 (0.02, 0.11)	**0.002**	-0.01 (-0.05, 0.04)	0.740	0.05 (0.00, 0.09)	**0.031**
TG	-0.03 (-0.11, 0.04)	0.368	0.03 (-0.04, 0.10)	0.360	-0.04 (-0.11, 0.03)	0.292	0.03 (-0.05, 0.10)	0.454	0.02 (-0.05, 0.10)	0.510
**Adjusted model +CHO (En%)**										
TC	-0.12 (-0.20, -0.05)	**0.002**	-0.05 (-0.12, 0.02)	0.168	-0.05 (-0.12, 0.02)	0.096	-0.03 (-0.11, 0.04)	0.403	0.09 (0.02, 0.17)	**0.014**
HDL-C	-0.03 (-0.05, -0.01)	**0.008**	-0.02 (-0.04, 0.00)	0.076	0.02 (0.00, 0.04)	0.078	-0.01 (-0.03, 0.01)	0.459	0.03 (0.01, 0.05)	**0.007**
LDL-C	-0.06 (-0.11, -0.02)	**0.007**	-0.03 (-0.08, 0.01)	0.116	0.04 (-0.01, 0.08)	0.134	-0.01 (-0.06, 0.03)	0.592	0.05 (0.01, 0.10)	**0.014**
TG	-0.05 (-0.12, 0.03)	0.232	0.04 (-0.03, 0.11)	0.265	-0.01 (-0.09, 0.06)	0.719	0.03 (-0.04, 0.11)	0.401	0.02 (-0.05, 0.09)	0.608
**Adjusted model +Pro (En%)**										
TC	-0.14 (-0.22, -0.07)	**<0.001**	-0.05 (-0.12, 0.03)	0.204	0.08 (-0.02, 0.17)	0.110	-0.02 (-0.10, 0.05)	0.535	0.09 (0.02, 0.17)	**0.016**
HDL-C	-0.03 (-0.05, -0.01)	**0.002**	-0.02 (-0.03, 0.00)	0.103	0.02 (0.00, 0.05)	0.078	-0.01 (-0.02, 0.01)	0.611	0.03 (0.01, 0.05)	**0.009**
LDL-C	-0.07 (-0.12, -0.03)	**0.001**	-0.03 (-0.07, 0.01)	0.152	0.04 (-0.01, 0.10)	0.125	-0.01 (-0.05, 0.04)	0.788	0.05 (0.01, 0.10)	**0.016**
TG	-0.04 (-0.11, 0.03)	0.293	0.04 (-0.03, 0.11)	0.241	-0.01 (-0.10, 0.09)	0.918	0.03 (-0.05, 0.10)	0.485	0.02 (-0.06, 0.09)	0.697
**Adjusted model +Pro /CHO(En%)**										
TC	-0.13 (-0.21, -0.06)	**0.001**	-0.05 (-0.12, 0.02)	0.159	0.06 (-0.03, 0.15)	0.195	-0.03 (-0.10, 0.05)	0.489	0.10 (0.02, 0.17)	**0.012**
HDL-C	-0.03 (-0.05, -0.01)	**0.003**	-0.02 (-0.04, 0.00)	0.073	0.02 (-0.01, 0.04)	0.157	-0.01 (-0.03, 0.01)	0.561	0.03 (0.01, 0.05)	**0.006**

LDL-C	-0.07 (-0.11, -0.02)	**0.003**	-0.04 (-0.08, 0.01)	0.106	0.03 (-0.02, 0.08)	0.273	-0.01 (-0.05, 0.04)	0.723	0.06 (0.01, 0.10)	**0.011**
TG	-0.04 (-0.12, 0.03)	0.242	0.04 (-0.03, 0.11)	0.219	0.00 (-0.09, 0.09)	0.966	0.03 (-0.05, 0.10)	0.444	0.01 (-0.06, 0.09)	0.708
**Adjusted model +total fat**										
TC	-0.13 (-0.21, -0.06)	**0.001**	-0.04 (-0.11, 0.03)	0.228	0.10 (0.03, 0.17)	**0.008**	-0.03 (-0.11, 0.05)	0.444	0.09 (0.01, 0.16)	0.021
HDL-C	-0.03 (-0.05, -0.01)	**0.003**	-0.01 (-0.03, 0.00)	0.110	0.03 (0.01, 0.05)	**0.005**	-0.01 (-0.03, 0.01)	0.505	0.02 (0.01, 0.04)	**0.011**
LDL-C	-0.07 (-0.11, -0.02)	**0.003**	-0.03 (-0.07, 0.01)	0.174	0.06 (0.02, 0.10)	**0.008**	-0.01 (-0.05, 0.03)	0.652	0.05 (0.01, 0.09)	**0.023**
TG	-0.04 (-0.11, 0.03)	0.298	0.04 (-0.03, 0.10)	0.317	-0.03 (-0.10, 0.04)	0.369	0.03 (-0.04, 0.10)	0.429	0.02 (-0.05, 0.09)	0.542
**Adjusted model +dietary cholesterol**										
TC	-0.15 (-0.22, -0.07)	**<0.001**	-0.04 (-0.11, 0.03)	0.301	0.07 (-0.03, 0.17)	0.175	-0.03 (-0.11, 0.05)	0.444	0.08 (0.00, 0.15)	**0.043**
HDL-C	-0.03 (-0.05, -0.01)	**0.001**	-0.01 (-0.03, 0.01)	0.155	0.02 (-0.01, 0.04)	0.248	-0.01 (-0.03, 0.01)	0.489	0.02 (0.00, 0.04)	**0.029**
LDL-C	-0.08 (-0.12, -0.03)	**0.001**	-0.02 (-0.07, 0.02)	0.246	0.03 (-0.03, 0.09)	0.263	-0.01 (-0.06, 0.03)	0.649	0.04 (0.00, 0.09)	0.055
TG	-0.03 (-0.11, 0.04)	0.373	0.03 (-0.04, 0.10)	0.352	-0.01 (-0.11, 0.09)	0.837	0.03 (-0.04, 0.10)	0.417	0.03 (-0.04, 0.10)	0.446
**Third trimester**										
**Adjusted model**										
TC	-0.17 (-0.25, -0.08)	**<0.001**	-0.03 (-0.11, 0.05)	0.476	0.14 (0.06, 0.23)	**0.001**	-0.05 (-0.14, 0.04)	0.242	0.12 (0.03, 0.20)	**0.011**
HDL-C	-0.05 (-0.07, -0.03)	**<0.001**	0.00 (-0.03, 0.02)	0.697	0.04 (0.02, 0.06)	**<0.001**	-0.01 (-0.04, 0.01)	0.215	0.03 (0.01, 0.05)	**0.013**
LDL-C	-0.10 (-0.16, -0.05)	**<0.001**	-0.01 (-0.07, 0.04)	0.572	0.09 (0.03, 0.14)	**0.001**	-0.04 (-0.09, 0.02)	0.173	0.07 (0.02, 0.12)	**0.012**
TG	-0.06 (-0.15, 0.04)	0.250	0.04 (-0.05, 0.13)	0.428	-0.05 (-0.14, 0.05)	0.340	-0.04 (-0.14, 0.05)	0.391	0.02 (-0.08, 0.11)	0.703
**Adjusted model**										

**+CHO (En%)**										
TC	-0.13 (-0.22, -0.04)	**0.005**	-0.05 (-0.14, 0.03)	0.216	0.08 (-0.02, 0.17)	0.122	-0.06 (-0.15, 0.02)	0.155	0.13 (0.04, 0.22)	**0.005**
HDL-C	-0.04 (-0.06, -0.02)	**0.001**	-0.01 (-0.03, 0.01)	0.359	0.02 (0.00, 0.05)	0.062	-0.02 (-0.04, 0.01)	0.133	0.03 (0.01, 0.05)	**0.006**
LDL-C	-0.08 (-0.14, -0.03)	**0.003**	-0.03 (-0.08, 0.02)	0.286	0.05 (-0.01, 0.11)	0.097	-0.04 (-0.10, 0.01)	0.106	0.08 (0.02, 0.13)	**0.006**
TG	-0.06 (-0.15, 0.04)	0.273	0.04 (-0.06, 0.13)	0.441	-0.05 (-0.15, 0.06)	0.378	-0.04 (-0.14, 0.05)	0.391	0.02 (-0.08, 0.12)	0.701
**Adjusted model +Pro (En%)**										
TC	-0.16 (-0.25, -0.07)	**<0.001**	-0.04 (-0.13, 0.05)	0.359	0.11 (0.00, 0.22)	0.057	-0.05 (-0.14, 0.04)	0.256	0.13 (0.03, 0.22)	**0.007**
HDL-C	-0.05(-0.07, -0.02)	**<0.001**	-0.01 (-0.03, 0.02)	0.590	0.03 (0.01, 0.06)	**0.019**	-0.01 (-0.04, 0.01)	0.225	0.03 (0.01, 0.05)	**0.011**
LDL-C	-0.10 (-0.15, -0.05)	**<0.001**	-0.02 (-0.07, 0.03)	0.422	0.06 (0.00, 0.13)	0.060	-0.04 (-0.09, 0.02)	0.186	0.07 (0.02, 0.13)	**0.008**
TG	-0.06 (-0.16, 0.04)	0.217	0.05 (-0.05, 0.14)	0.347	-0.02 (-0.14, 0.10)	0.768	-0.04 (-0.14, 0.05)	0.378	0.01 (-0.09, 0.11)	0.829
**Adjusted model +Pro /CHO(En%)**										
TC	-0.15 (-0.24,-0.06)	**0.001**	-0.05 (-0.14, 0.03)	0.240	0.07 (-0.03, 0.18)	0.182	-0.05 (-0.14, 0.03)	0.223	0.13 (0.04, 0.22)	**0.004**
HDL-C	-0.04 (-0.07, -0.02)	**<0.001**	-0.01 (-0.03, 0.01)	0.397	0.02 (0.00, 0.05)	0.099	-0.01 (-0.04, 0.01)	0.198	0.03 (0.01, 0.06)	**0.005**
LDL-C	-0.09 (-0.14, -0.04)	**0.001**	-0.03 (-0.08, 0.02)	0.288	0.04 (-0.02, 0.11)	0.191	-0.04 (-0.09, 0.02)	0.157	0.08 (0.03, 0.13)	**0.004**
TG	-0.06 (-0.15, 0.04)	0.248	0.04 (-0.06, 0.13)	0.421	-0.04 (-0.16, 0.08)	0.501	-0.04 (-0.14, 0.05)	0.394	0.02 (-0.08, 0.12)	0.726
**Adjusted model + total fat**										
TC	-0.14 (-0.23, -0.05)	**0.002**	-0.04 (-0.13, 0.04)	0.298	0.12 (0.04, 0.21)	**0.006**	-0.06 (-0.15, 0.03)	0.184	0.12 (0.03, 0.21)	**0.008**
HDL-C	-0.04 (-0.06, -0.02)	**<0.001**	-0.01 (-0.03, 0.01)	0.466	0.03 (0.01, 0.06)	**0.002**	-0.02 (-0.04, 0.01)	0.156	0.03 (0.01, 0.05)	**0.010**
LDL-C	-0.09 (-0.14, -0.04)	**0.001**	-0.02 (-0.07, 0.03)	0.386	0.08 (0.02, 0.13)	**0.004**	-0.04 (-0.09, 0.01)	0.128	0.07 (0.02, 0.12)	**0.010**

TG	-0.05 (-0.15, 0.04)	0.295	0.03 (-0.06, 0.12)	0.464	-0.05 (-0.14, 0.05)	0.312	-0.04 (-0.14, 0.05)	0.379	0.02 (-0.08, 0.12)	0.694
**Adjusted model +dietary cholesterol**										
TC	-0.17 (-0.25, -0.08)	**<0.001**	-0.03 (-0.11, 0.05)	0.473	0.08 (-0.04, 0.19)	0.208	-0.06 (-0.15, 0.03)	0.193	0.11 (0.02, 0.20)	**0.018**
HDL-C	-0.05 (-0.07, -0.03)	**<0.001**	0.00 (-0.03, 0.02)	0.699	0.02 (-0.01, 0.05)	0.164	-0.02 (-0.04, 0.01)	0.163	0.03 (0.00, 0.05)	**0.023**
LDL-C	-0.10 (-0.16, -0.05)	**<0.001**	-0.01 (-0.06, 0.04)	0.574	0.05 (-0.02, 0.12)	0.178	-0.04 (-0.09, 0.01)	0.135	0.06 (0.01, 0.12)	**0.020**
TG	-0.06 (-0.15, 0.04)	0.251	0.04 (-0.05, 0.13)	0.428	-0.04 (-0.17, 0.09)	0.537	-0.04 (-0.14, 0.05)	0.399	0.02 (-0.08, 0.12)	0.695

TC total cholesterol, HDL-C high-density lipoprotein cholesterol, LDL-C low-density lipoprotein cholesterol, TG triacylglycerol

Adjusted model was adjusted for maternal age, Pre-BMI, ethnology, parity, education, household income, smoking and passive smoking, alcohol drinking, pre-pregnancy BMI, and first-degree family history of diabetes. MET-min/W, other dietary patterns, energy intake, GWG and gestational weeks.

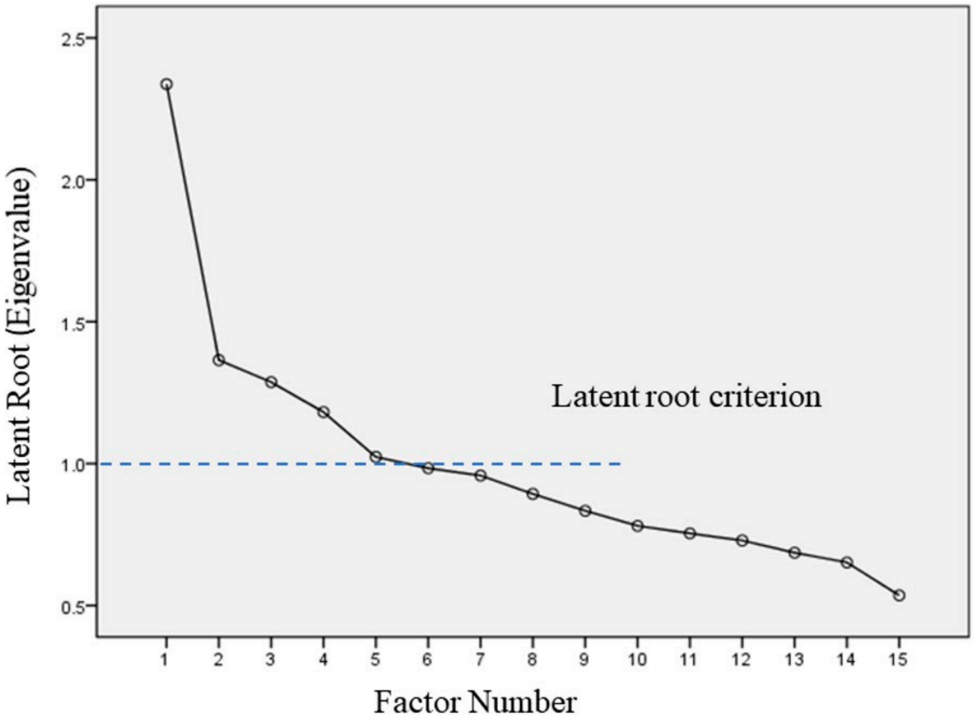

Figure S1. Scree plot for identification of dietary patterns by principal component analysis.
